# Biomechanical Substantiation of the Strength and Stiffness of a Hip Joint Capsule Defect Fixation with Polypropylene Mesh

**DOI:** 10.1055/s-0039-3402793

**Published:** 2020-01-21

**Authors:** Serhii Olegovich Maslennikov, Serhii Pavlovich Panchenko, Maxim Leonidovich Golovakha

**Affiliations:** 1 Department of Traumatology and Orthopaedics, Zaporizhia State Medical University, Zaporozhye, St. Steshenko, Ukraine; 2 Department of Structural Mechanics and Strength of Materials, State Higher Educational Institution, Prydniprovska State Academy of Civil Engineering and Architecture, Dnipro, Ukraine

**Keywords:** endoprosthesis, hip joint, modeling, polypropylene, total hip arthroplasty

## Abstract

**Formulation of the problem** Dislocation of the femoral component of the endoprosthesis is one of the most frequent complications of total hip replacement. The best option for the “treatment” of dislocation of the hip endoprosthesis is to prevent the development of primary instability. There are cases in which even with the correct installation of the endoprosthesis components, dislocations arise due to the weakness or defect of the capsular–ligament apparatus. Currently, many methods have been developed to strengthen and restore the posterior structures of the capsule of the hip joint with the help of auto- and allomaterials, which differ in both the fixation technique and the characteristics of the materials themselves. In this paper, we propose a method for restoring and strengthening the posterior structures of the capsule of the hip joint using polypropylene-based graft implants. The purpose of this study is to, with the help of specialized software, build a model of the capsule of the hip joint after capsulotomy and to determine the stiffness capabilities of the defect covered with polypropylene mesh.

**Results** The study was performed using a software package based on the finite-element method. As a result of the performed calculations, pictures of the distribution of the stress–strain state in the “head-capsule” system were obtained. To assess the effectiveness of the method of closing the capsule, from the viewpoint of rigidity, as the main characteristics, the values of the opening of the cut are selected.

**Conclusions** Under the kinematic loading of the model, the smallest values of the opening of the section are obtained when it is closed by a grid. In the case of thread fixation, the values were higher by 8.5%. However, the values of equivalent stresses, both in the capsule and in the head, in the model with the grid turned out to be the largest. These stresses were higher by 23.8% in the capsule and by 60.4% in the head than the same values for the thread fixation model. The obtained results indicate that the model with a grid is more rigid in the considered fixation variants.

Dislocation of the femoral component is one of the most frequent complications of total hip arthroplasty (THA). There are many factors contributing to the development of dislocations, which can be grouped into three main categories: patient-dependent factors, implant-dependent factors, and factors associated with operative technique. 1 The best option in the “treatment” of a THA dislocation is to prevent the development of primary instability. 2 3 However, there are cases in which even with the proper installation of the endoprosthesis components, the dislocation results from weakness or a defect in the capsule–ligament apparatus; for example, it can develop during repeated revision operations, or the consequences of severe injuries of the proximal femur can lead to the excision of massively expanded scar tissue. The dislocation or dysplastic changes can also occur after operations which require limb lengthening and so on.

The issue of the restoration of the capsule–ligament apparatus is particularly relevant in people with obesity, which is clinically characterized by the deposition of fat in different parts of the body, including the thighs and anterior abdominal wall. Thus, due to excessive deposition of adipose tissue, during the squat, a mechanical disturbance occurs and an additional fixation point is created, around which additional force arises, which can be significant and in some cases reach 20% of body weight. 4 It affects the kinematics of movement with full flexion and increases the risk of dislocation of the endoprosthesis head from the acetabulum cup ( Fig. 1 ).

**Fig. 1:**
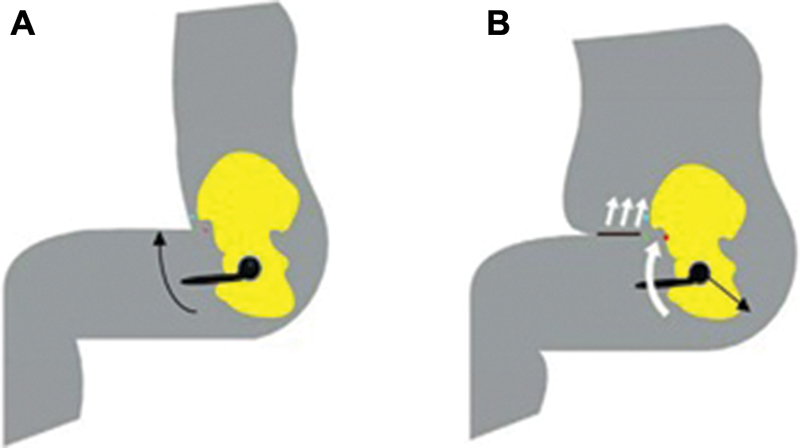
Development of a hip joint endoprosthesis dislocation in obese patients. ( **A** ) A sagittal plane at the time of maximum flexion in a patient not suffering from obesity. ( **B** ) Unfavorable kinematics in a patient with obesity, with the occurrence of an additional point of fixation followed by a torque of the head of the prosthesis, leading to a posterior hip dislocation (Elkins Jacob Matthias, University of Iowa, 2013).

In the case of posterolateral approaches, careful restoration of the posterior structures of the capsule and external rotators (tendons of the piriformis muscle) with nonabsorbable sutures is one of the main conditions, though it is not always applicable. Many authors argue that the careful restoration of soft tissue structures or, at least, the preservation of these structures using modified approaches significantly reduces the incidence of dislocations associated with posterolateral surgical approach. Therefore, it was estimated that with the approach without the restoration of soft tissues, the risk of dislocation is 8.21 times higher than with the same approach but with the restoration of soft tissues. 5 In addition, the dependence of the hip endoprosthesis stability on the capsule thickness has been proven. A joint capsule with a thickness of 1 mm weathers two times less stress, leading to dislocation than a joint capsule with a thickness of 3.5 mm, and three times less with a maximum capsule thickness of 6 mm. 6 It should be also noted that suturing even with significant damage to the capsule by a longitudinal incision successfully restores stability to approximately 10% of the baseline. Thus, the proven importance of the restoration and strengthening of the structures of the capsule–ligament apparatus is in line with methods of preventing the development of dislocation of the hip endoprosthesis, such as careful preoperative planning, 7 correct installation of components, and patient management in the postoperative period.

Currently, many methods have been developed to strengthen and restore the posterior structures of the hip joint capsule using auto- and allomaterials, which differ both in the method of fixation and in the characteristics of the materials themselves. Alongside their advantages, these methods are characterized by several disadvantages such as high manufacturing cost and the need for special skills of the operating surgeon. We have proposed a method of restoration and strengthening of the posterior structures of the hip joint capsule with polypropylene mesh (PPM) implants. 8 The essence of the proposed method lies in the fact that having all the components of the hip joint endoprosthesis installed, PPM is applied in the projection of the capsule defect on its outer surface while its edges are hemmed to the free edges of the capsule, along its entire perimeter, over the entire thickness of the capsule, thus forming a mechanical “patch” over the defect and acting as a plateau for the formation of a durable fibrous scar. This method does not require significant financial costs and/or special skills of the surgeon and demonstrates convincing results proved by histological studies on laboratory animals (the study was conducted in accordance with the Law of Ukraine on “Scientific and Scientific and Technical Activities” and the Council of Europe's European Convention for the Protection of the Vertebrate Animals used for Experimental and Other Scientific Purposes [Strasbourg, 1985]); bioinertness and the safety data are based on this experiments. Moreover, during the experimental study, physical and mechanical properties (using Young's modulus and Poisson's ratio) of PPM with soft tissues integrated into it were collected. A computer model was built and further calculations were performed with regard to the obtained information.

The aim of the study is to assess the strength and stiffness of the closure of the hip joint capsule defect with PPM based on an analysis of the stress–strain condition of the capsule models sutured by various methods.

## Materials and Methods

The study was performed using a software package “ANSYS” 18.2 based on the finite-element method. For the rational use of computer resources, a computational model was built, which consisted of a capsule model and a model of endoprosthesis head. The bones that form the hip joint were not modeled, and their presence was taken into account by applying appropriate boundary conditions; moving the edges of the capsule in all directions was forbidden.

**Fig. 2:**
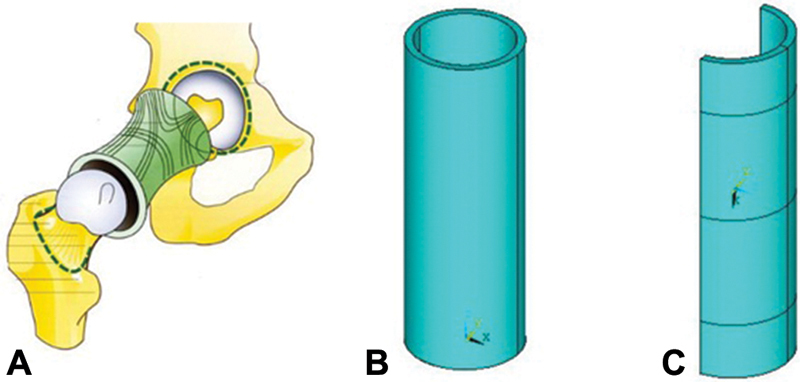
( **A** ) Hip joint capsule. ( **B,C** ) The “capsule” element of the computer model of the “capsule–head of the hip joint endoprosthesis” system.

The model of the capsule in its size corresponded to the anatomical size of the hip joint capsule of an adult. Considering that the capsule shape follows the contours of the femoral head and neck and has the shape of a cylindrical sleeve attached to the edges of the acetabulum and intertrochanteric line ( Fig. 2A ), it was modeled as a hollow cylinder with the following dimensions: length of 12 cm with wall thickness of 3 mm ( Fig. 2B ). 9 Regarding the diameter of the cylinder, the following should be noted. Considering the fact that during the movements, the surface of the endoprosthesis head impacts the capsule, to comply with the conditions of interaction between these elements, the diameter of the head and the internal diameter of the cylinder had the same size, which was 36 mm. 10 Taking into account the specified wall thickness of the capsule, the outer diameter of the cylinder was 42 mm. To reduce the number of finite elements of the model, only 1/2 cylinder was considered ( Fig. 2C ).

**Fig. 3:**
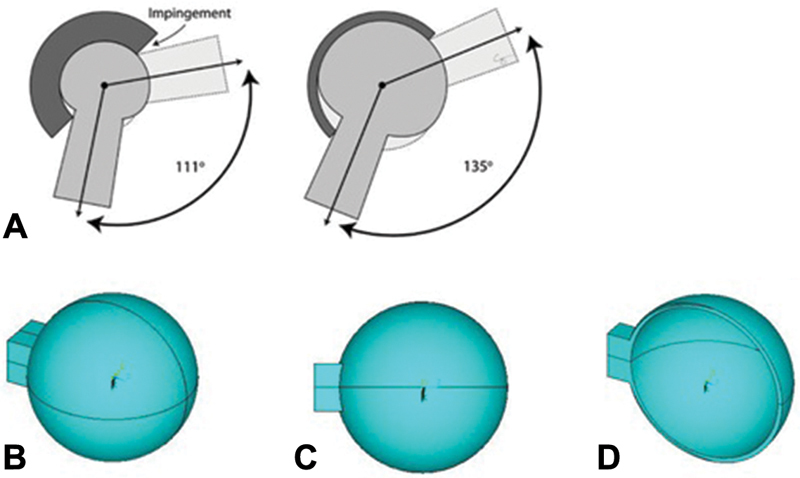
( **A** ) Location of the endoprosthesis head in the acetabular component. ( **B–D** ) The “endoprosthesis head” element of the computer model of the “capsule–head of the hip joint endoprosthesis” system.

In regard to the head model, we note that its rigidity is much higher than that of the capsule. In addition, the endoprosthesis is not a direct object of investigation. Therefore, to reduce the total number of finite elements in the model, the head of the hip joint endoprosthesis was modeled as a hollow sphere, the outer diameter of which, as mentioned previously, is 36 mm in size and 34 mm in internal diameter, that is, the head thickness is 1 mm ( Fig. 3 ). To apply a load to the head of the endoprosthesis, which is later transferred to the capsule, a rectangular element is attached to the sphere, which has a cross-section of 1 × 1 cm.

The interaction between the sphere and the inner surface of the cylinder was performed by creating a contact pair using software tools.

Two ways of capsule suturing were investigated in this study: interrupted stitches and suturing with PPM, which covered the defect of the capsule and fixed to it along the entire perimeter of the mesh through the entire capsular-ligament apparatus. Consequently, two computational models were built. While the geometry of the two was the same, they differed in the method of closing the dissected capsule. An additional control model with the identical dimensions was constructed, yet the incision was not sutured here.

Capsulotomy was modeled as a cut of 0 thickness, along the basic cylinder, that is, along the capsule model. The length of the incision is 8 cm. The incision was located symmetrically along the height of the cylinder; therefore, the shift from the upper and lower bases of the model is 2 cm.

**Fig. 4:**
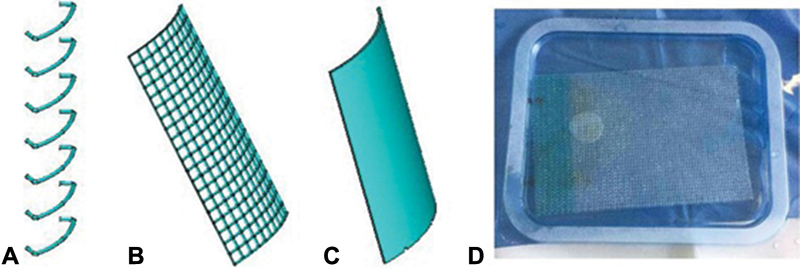
( **A** ) the element “interrupted stitches.” ( **B,C** ) The element “polypropylene mesh” of the computer model of the “capsule–head of the hip joint endoprosthesis” system. ( **D** ) The polypropylene mesh used in the experiment.

The locking elements (thread and mesh) were also modeled in accordance with their actual dimensions ( Fig. 4A, B ). The thread diameter is 0.5 mm, the thread diameter in the mesh is 0.5 mm, and the size of the cells is 2 × 2 mm.

The physical and mechanical properties of the model elements are as follows. For the capsule–ligament apparatus, Young's modulus of elasticity is 150 MPa, and Poisson's ratio is 0.25. The properties of the endoprosthesis head were chosen because of high rigidity compared with the capsule rigidity, which amounted to 2 × 105 and 0.25 MPa, respectively. For the thread and the mesh, the elastic properties were assumed to be the same and corresponding: Young's modulus value was 17.2 MPa, the Poisson ratio was 0.25. However, it should be noted that a preliminary calculation showed that modeling the mesh with its real dimensions (cell structure) creates a large number of additional elements in the model (lines and surfaces). Each of these elements was assigned a number. This, in turn, leads to a significant consumption of computer resources. Therefore, the mesh model was replaced with a fragment of a cylindrical surface ( Fig. 4C ), the overall dimensions of which corresponded to the mesh, and the thickness coincided with the thread diameter. This replacement required recalculation of the elastic modulus, which amounted to 1.72 MPa. Note that the mesh modeling in the form of a solid surface is legitimate since the difference in the structure of these objects exists only (the presence or absence of cells). At the macro level, by defining a new modulus of elasticity for a continuous surface, there will be no difference in the behavior of mesh models (cell or solid structure) when it is loaded. Consequently, the impact on the capsule from the side of the mesh model in the form of a solid surface will be the same as in the simulation of its cell structure.

**Fig. 5:**
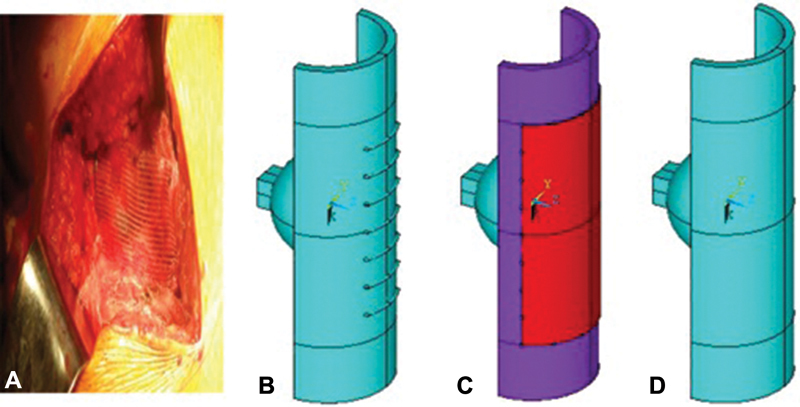
( **A** ) Polypropylene mesh implanted to close the capsular defect. ( **B** ) Computer model of the “capsule–head of the hip joint endoprosthesis” with the closure of the defect of the capsule with an anchor stitch. ( **C** ) Polypropylene mesh. ( **D** ) Computer model of the “capsule–head of the hip joint endoprosthesis” system without the closure of the defect.

Closure of the defect was simulated by stitching and the mesh. The sutures were placed with a step of 1 cm at a distance of 1 cm from the edges of the cut, and therefore the total number of stitches was seven ( Fig. 5B ). The shifts from the axis of the incision were also 1 cm each. The sizing of the mesh corresponded to the sizes of the cut, that is, 8 cm along the cut line, and with the intent of 2.5 cm from its axis ( Fig. 5C ). The mesh and the surface of the capsule were connected by cylindrical elements that simulated sewing. The dimensions and properties of the cylindrical elements corresponded to the sizes and properties of the thread used to sew the mesh to the capsule and were applied with a step of 1 cm along the perimeter of the mesh.

Another contact pair was created to connect the locking elements and the capsule. To study the strength and stiffness of the dissection fixation in various ways, two types of loads were applied to a rectangular element of the head model: static, in the form of a fixed force, and kinematic, in the form of a fixed displacement. In all cases, the vector of the load application was directed along the normal to the cutting line. Given that the load on the center of the incision is considered as the most dangerous effect, the study was performed under the assumption that the head is located symmetrically with respect to the incision line both in length and axis.

The amount of movement of the endoprosthesis head in the direction of the incision was chosen from the assumption that one-fourth of the head diameter makes 9 mm. The magnitude of the static force was 15 kg. It is noteworthy that since under all the same conditions the models differ only in the methods of fixation, the magnitude of the loads can be chosen arbitrarily to compare their effectiveness.

The capsule model was fixed along the entire plane of the upper and lower bases; movement in all directions which imitated the attachment of the capsule to the bone surface was prohibited. To this, the corresponding boundary conditions were superimposed on the edges located on the side of the rejected part of the cylinder, which ensured the immobility of the indicated edges of the model in the direction of the load vector.

Computing of finite elements was performed by the generator of grids of the software complex. The element type selected was *solid* . The size of the finite element was set on the lines of the objects and varied from 0.25 to 1.0 mm. The created contact pair, “capsule–head of the hip joint endoprosthesis,” suggested the absence of friction.

## Results and Discussion

Images of the distribution of the stress–strain state in the “head–capsule” system were calculated. To assess the effectiveness of the method of the closure of the capsule with regard to stiffness as the main characteristics, the values of the opening of the incision, as well as stresses arising in the joint capsule, were defined. The stress in the head is considered as an additional characteristic. The results are shown in the respective tables ( Tables 1 – 3 ).

Note that at the first stage of the study, a kinematic calculation was performed, which is aimed at studying the rigidity of fixation. Since the specified displacement of the head will be the same for all models, the opening of the cut may also be the same or similar in values for different models. An indicator of stiffness will be the magnitudes of the stresses arising in the model since the more rigid model is more resistant to the applied loads, which manifests itself in an increase in stresses.

Calculations have shown that for a given loading pattern, the largest displacements occur in the center of the section, that is, in the place of the impact of the head on the capsule. In this case, the maximum amount of movement does not occur on the cutting line but is displaced from it in the circumferential direction, which is associated with the deformation of the capsule. Therefore, this value cannot be the main indicator of disclosure. Thus, to estimate the magnitude of the disclosure, displacements of points located on the incision line inside ( *δ*_ís_ ) and outside ( *δ*_os_ ) of the capsule were used.

Table 1 demonstrates the way in which the method of suturing the incision influences the values of maximum displacements ( *δ*_max_ ) occurring in the capsule, which are directed toward the opening, that is, around the circumference of the cylinder. It also presents the movements of points located on the surface of the section in the same direction ( *δ*_is_ , *δ*_os_ ), that is, deviations of the edges of the incision from its line.

**Table 1:** The values of the displacements and stresses in the system “capsule–head of the hip joint endoprosthesis” with the kinematic calculation

Fixation method	Displacement, mm			Stress σ _Miz_ , MPa	
	*δ*_max_	*δ*_is_	*δ*_os_	Capsule	Head
Model without closing capsulotomy (control model)	6.97	5.79	6.97	20.9	27.4
Model of the system with the closure of the defect with sutures	6.35	5.37	6.04	26.9	54.5
Model of the system with the closure of the defect with polypropylene mesh	5.85	4.94	5.72	33.3	87.4

**Fig. 6:**
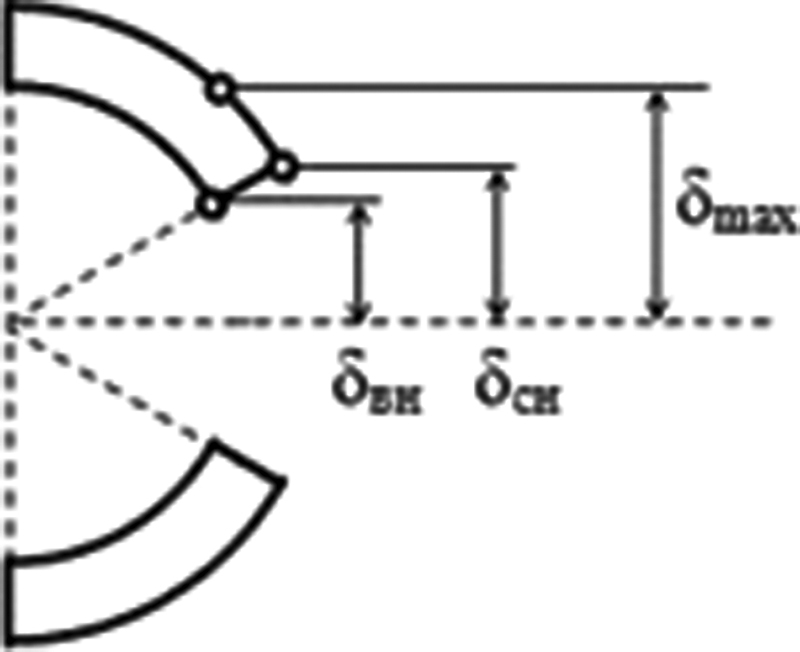
The location of the points of the capsule with the largest displacements.

As can be seen in Table 1 , the values of movements at the edges of the incision inside and outside the capsule are different. Moreover, the amount of movement outside is greater than that on the inner surface, that is, there is a reversal of the edges of the cut. The reversal pattern is shown in Fig. 6 .

Analysis of the results given in Table 1 showed the following. The smallest displacements in the direction of opening were obtained from the model of fixation of the incision with PPM and amounted to 5.85 mm. In case the incision was fixed with a suture, this value was 6.35 mm, which was to be 0.5 mm or 8.5% higher. The largest displacements were obtained for the control model (without fixation), which were equal to 6.97 mm, and were larger than the models with fixation with threads and a mesh of 1.12 mm or 19.1% and 0.62 mm or 9.8%, respectively. Regarding the deviation values, it can be noted that in the model of fixation with the mesh, it turned out to be also the smallest both on the inside and on the outside. The difference in these values was 1.56 mm. In the fixation model, the thread deviation was higher by 8.7% from the inside and by 5.6% from the outside, and the difference was 1.34 mm. In the control model, deviations were greatest and exceeded these indicators by 17.2% from the inside and by 21.9% from the outside for the model with a mesh and by 7.8% from the inside and 15.4% from the outside for the model with a thread. The difference in deviations for the control model was 2.36 mm.

Given that *δ* is the deviation of the points of the capsule from the axis of the incision, full disclosure is determined from the following correlation:

Δ = 2* *δ* .

Thus, the full values of disclosures are given in Table 2 .

**Table 2:** The size of the opening of the incision depending on the model of fixation in the kinematic calculation

Fixation method	Displacement, mm		
	Δ _max_	Δ _is_	Δ _os_
Model without closing capsulotomy (control model)	13.94	11.58	13.94
Model of the system with the closure of the defect with sutures	12.7	10.74	12.08
Model of the system with the closure of the defect with polypropylene mesh	11.7	9.88	11.44

**Fig. 7:**
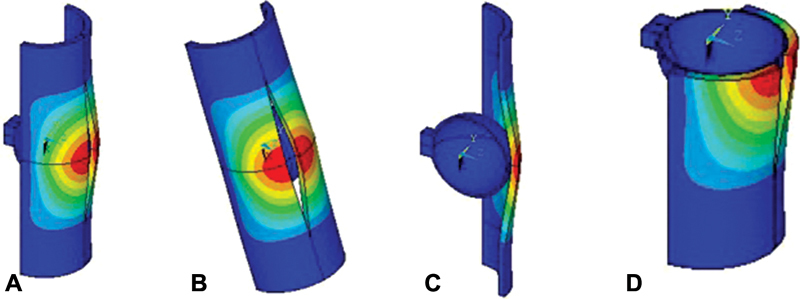
The distribution of deformations in the “capsule–head of the hip joint endoprosthesis” model.

To visualize the calculations, Fig. 7 shows patterns of the distribution of displacements in the model in the circumferential direction. The species are given in full ( Fig. 7A, B ), as well as in the longitudinal ( Fig. 7C ) and transverse ( Fig. 7D ) sections in relation to the cut line. Only the capsule and the head without the fixing element are shown. Note that the distribution pattern of these movements is the same regardless of the fixation model; therefore, in Fig. 7 , for the type of the control model is shown (without fixation of the incision).

To assess the stress in the “capsule–head of the hip joint endoprosthesis” system, the values of Mises equivalent stresses both in the capsule and in the head were defined.

**Fig. 8:**
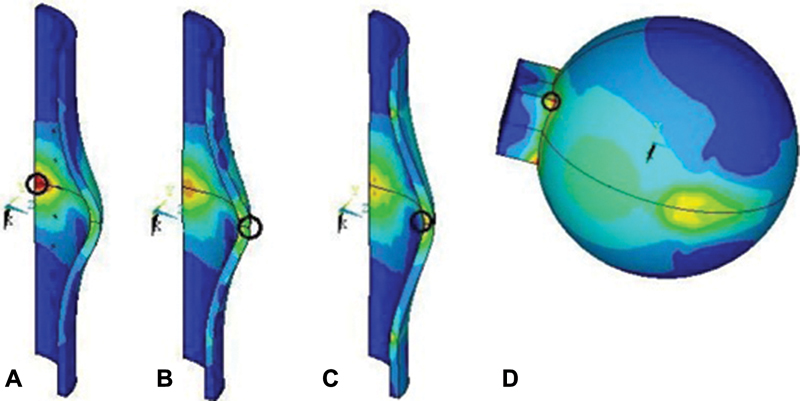
Stress distribution in the “capsule–head of the hip joint endoprosthesis” model. ( **A–C** ) In the capsule. ( **D** ) In the head.

Table 1 demonstrates that the greatest stress in the capsule develops in the model with mesh fixation on the line of intersection of the transverse plane of the model symmetry and the longitudinal edge of the model of the capsule from the inside ( Fig. 8A ). The magnitude of these stresses was 33.3 MPa. In case of the fixation with a thread, the magnitude of these stresses was 19.2% less and equaled to 26.9 MPa. The stress also developed in the transverse plane of the model symmetry but in the center of the section from the outside ( Fig. 8B ). The lowest loads were obtained in the control model (20.9 MPa) and were 37.2% less compared with the model with a mesh and 22.3% less than the model with a thread. These stresses appeared in the center of the section from the inside of the model ( Fig. 8C ).

The magnitudes of the maximum stress in the head were distributed between the models in the same way. The major stress was fixed in a model with the mesh and peaked 87.4 MPa. In the model with the thread fixation, the stress was 37.6% less. And the smallest values were obtained from the model without fixation, which is 68.6 and 49.7% less compared with the models with mesh and thread fixation methods, respectively. The indicated stress developed at the point of the ball's connection with the parallelepiped ( Fig. 8 ), that is, in the stress concentrator, which explains their value.

As mentioned previously, the kinematic calculation proved that the stress distribution indicates the rigidity characteristics of the studied models. The higher the stress under the same load conditions, the more rigid the model. The obtained values show that in regard to fixation rigidity, the model with the mesh is more rigid.

At the next stage of the work, a static calculation was performed, that is, the magnitude of the applied load was recorded. It is noteworthy that in case of a fixed amount of force, models with different stiffness will also have different displacements—the harder the model, the less displacement. Therefore, the main indicator of this study is the size of the opening of the section, and the stress values of the model can be used to further assess the strength.

Analysis of the results in Table 3 showed that the smallest displacements in the direction of opening were obtained from the model of fixation of the incision with a mesh and amounted to 1.01 mm. When the incision was fixed with a thread, this value was 1.68 mm, which turned out to be higher by 0.67 mm or 66.3%. The largest displacements were obtained from the control model (without fixation), which were equal to 3.42 mm, and were larger than the models with fixation by threads and mesh by 1.74 mm or 103.6% and 2.41 mm or 238.6%, respectively.

**Table 3:** The magnitude of the displacements and stresses in the system “capsule–head of the hip joint endoprosthesis” with a static calculation

Fixation method	Displacement in the capsule *δ* , mm			Stress σ _Miz_ , MPa		Head displacement *w* , mm
	*δ*_max_	*δ*_is_	*δ*_os_	Capsule	Head	
Model without closing capsulotomy (control model)	3.42	2.87	3.42	10.5	13.6	3.90
Model of the system with the closure of the defect with sutures	1.68	1.43	1.60	8.12	13.8	2.06
Model of the system with the closure of the defect with polypropylene mesh	1.01	0.86	0.99	7.59	13.5	1.38

It is noteworthy that in the kinematic calculation as well, the values of displacements at the edges of the incision inside and outside the capsule differ ( Table 3 ).

Taking into account the ratio of 2 * *δ* ( *δ* is the deviation of the points of the capsule from the axis of the incision), we obtain the full disclosure of the incision, which is shown in Table 4 .

**Table 4:** Values of incision disclosure depending on the model fixing during the static calculation

Fixation method	Displacement, mm		
	Δ _max_	Δ _is_	Δ _os_
Model without closing capsulotomy (control model)	6.84	5.74	6.84
Model of the system with the closure of the defect with sutures	3.36	2.86	3.20
Model of the system with the closure of the defect with polypropylene mesh	2.02	1.72	1.98

Regarding the magnitudes of the disclosure, it can be noted that in the model of fixation with the mesh, it was also the smallest both on the inside and on the outside. The difference in these values is 0.26 mm. In the fixation model, the opening was higher by 66.3% from the inside and by 61.6% from the outside, and the difference between them was 0.34 mm. In the control model, the disclosures were greatest and exceeded these indicators by 233.7% from the inside and 245.5% from the outside for the model with a grid and by 100.7% from the inside and 113.8% from the outside for the model with a thread. The difference in the disclosures for the control model was 1.10 mm.

It should be noted that in Table 3 , in addition to the values described previously and used to assess the effectiveness of the method for closing the capsule (displacement/stresses in the capsule and head), the magnitudes of displacement of the head also depend on the method of fixation. This is because when a fixed force is applied to the head, the movements in the model are not controlled but are the result. Therefore, the opening values are the indicator of not only stiffness but also the amount of movement of the head in the direction of the cut ( *w* = displacement in the direction of the applied force).

Table 3 shows the smallest movements in the model of fixation with a mesh, which is 1.38 mm. In the model of fixation with thread, this value was equal to 2.06 mm, which is 49.3% more. The largest displacements of the head obtained for the model without fixation, which equaled 3.90 mm, which larger than the model with the mesh by 182.6 and 89.3% than the model with the thread, respectively.

**Fig. 9:**
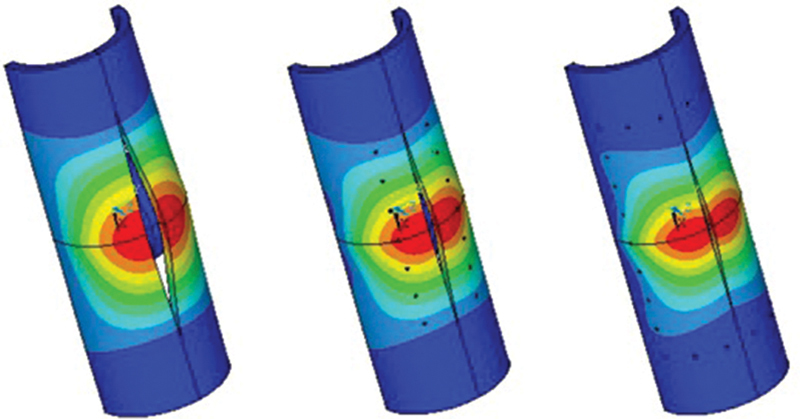
Deformations of the “capsule–head of the hip joint endoprosthesis” system in static calculation.

Note that displacements in models under static load are distributed in the same way as in the kinematic calculation ( Fig. 7 ). However, the magnitudes of displacements in the static calculation differ significantly for different models. Therefore, Fig. 9 shows patterns of the distribution of displacements in the circumferential direction for each model. To compare the magnitudes of the deformations of the models, the images are shown on the same scale. As can be seen from Fig. 9 , the smallest disclosure is fixed in the mesh fixation model ( Fig. 9C ), while fixation with a thread is medium ( Fig. 9B ), and the largest values are fived in the model without fixation ( Fig. 9A ).

To estimate the stress state, Mises equivalent stresses both in the capsule and in the head were selected.

Table 3 shows the greatest stresses in the capsule arise in the model without fixation, which, in magnitude, equaled 10.5 MPa. During fixation with a thread, these stresses are 22.7% less and amounted to 8.12 MPa. The lowest stresses occurred in the mesh fixation model (7.59 MPa) and are less by 27.7% than the control model and 6.53% less than the model with the thread. These stresses arose, in all models, at the point of contact between the head and the capsule.

The maximum stresses in the head are not practically differing in magnitude for different models of fixation. The largest of this stress is achieved with the thread model and amounted to 13.8 MPa. The model without latching stress is less by 1.45%. And the smallest ones are obtained for a model with a mesh, which is smaller than that of models with thread fixing and control by 2.17 and 0.74%, respectively. The indicated stresses appeared, as in the kinematic calculation, at the point of the sphere's connection of the head with the parallelepiped ( Fig. 8 ), that is, in the stress concentrator.

As mentioned previously, in a static calculation, the distribution of displacements indicates the rigidity characteristics of the models under consideration. The lower the displacement under the same loading conditions, the more rigid the model. The resulting movements show that in terms of rigidity of fixation, the model with the mesh is more rigid. In addition, the magnitude of the resulting stresses in the capsule indicates that from the point of view of strength, the mesh model is also more durable.

## Conclusions

- The smallest opening of the section is obtained in the model of fixation with a mesh, with both the kinematic and the static type of calculation.
- In the kinematic calculation, the value of Mises equivalent, both in the capsule and in the head, in the model with the mesh turned out to be the highest.
- The displacement of the head in the direction of the applied load, with a static type of load, is also the smallest in the model of hip joint capsule closure defect with mesh.
- The results of the calculations indicate that with the considered options for fixing the cut, both in terms of rigidity and strength, the model of fixation with a grid is more effective.
- Thus, the application of a PPM to close and strengthen the posterior structures of the joint provides for keeping the head in the endoprosthesis cup, which can be an additional factor for the reduction of dislocation risk in the hip joint endoprosthesis.
